# Public policy on Human Papillomavirus immunization: an analysis based on the Walt and Gilson policy triangle

**DOI:** 10.1590/1980-220X-REEUSP-2025-0521en

**Published:** 2026-08-14

**Authors:** María Angélica Saldías-Fernández, Jéssica García-Gutiérrez

**Affiliations:** 1 Universidad de Chile, Facultad de Medicina, Departamento de Enfermería, Santiago, Chile.

**Keywords:** Vaccines, Human Papillomavirus 31, Policy, Planning and Management, Universal Health Care, Nursing Care, Health Policy, Vacinas, Papillomavirus Humano 31, Políticas, Políticas, Planejamento e Administração em Saúde, Assistência de Saúde Universal, Cuidados de Enfermagem, Política de Saúde

## Abstract

**Objective:**

To examine and analyze the Human Papillomavirus vaccination public policy implemented in Chile from the Walt and Gilson health policy triangle’s perspective.

**Method:**

A scoping review was conducted according to JBI Manual for Evidence Synthesis guidelines to identify the following broad domains: actors, content, context, and process.

**Results:**

Of the 24 selected articles and official reports, nursing professionals were identified as key actors in a privileged position to increase public acceptance, vaccination coverage, and access. Regarding content, context, and process, new strategies are needed to enhance acceptance of vaccination in dynamic settings and to formulate effective evidence-based vaccination policies.

**Conclusion:**

Public policy analysis, mediated by the Walt and Gilson health policy triangle, allows for the identification of interactions among its components and clarifies the challenges associated with the promotion and prevention of diseases linked to the Human Papillomavirus, thereby contributing to the strengthening of collective health, healthcare systems, and global priorities aimed at comprehensive and sustainable human development.

## RESUMO

**Objetivo:**

Examinar e analisar a política pública de vacinação contra o Papilomavírus Humano implementada no Chile, sob a perspectiva do triângulo de análise de políticas de saúde de Walt e Gilson.

**Método:**

Revisão de escopo, estruturada de acordo com as diretrizes do Manual de Síntese de Evidências do JBI, para identificar as áreas gerais de atores, conteúdo, contexto e processo.

**Resultados:**

Dos 24 artigos e relatórios oficiais selecionados, os profissionais de enfermagem foram identificados como atores em posição privilegiada para aumentar a aceitação pública, a cobertura e o acesso à vacinação. Em relação ao conteúdo, contexto e processo, novas estratégias são necessárias para ampliar a aceitação do processo, considerando a presença de ambientes dinâmicos, e para formular políticas de vacinação eficazes e baseadas em evidências.

**Conclusão:**

A análise das políticas públicas, mediada pelo triângulo de Walt e Gilson, permite identificar as interações entre seus componentes e esclarecer os desafios relacionados à promoção e prevenção de doenças associadas ao Papilomavírus Humano, contribuindo para o fortalecimento da saúde coletiva, dos sistemas de saúde e das prioridades globais orientadas para o desenvolvimento humano integral e sustentável.

## INTRODUCTION

Cervical cancer (CC) constitutes a public health problem in low- and middle-income countries^(1,2)^ concentrated in regions such as Asia, Africa, and Latin America^(3)^. In high-income countries, incidence and mortality have declined over the past 20 years, a trend associated with preventive strategies and early case detection^(4)^. In Chile, this type of cancer is the third most common among women and directly affects their productive and reproductive stages^(4)^. In 2022, 646 women died from CC, resulting in an adjusted mortality rate of 6.07 per 100,000 inhabitants^(5)^.

One of the primary etiological factors for CC is the Human Papillomavirus (HPV)^(6)^, which has the pathophysiological capacity to cause a long-term asymptomatic infection that interferes with cellular proliferation and differentiation in the cervix, leading to lesions with neoplastic and invasive characteristics^(7)^. The literature indicates that the development of CC is driven by social and contextual factors^(8)^ that manifest as profound inequities in access to prevention mechanisms, as well as in timely screening, diagnosis, and treatment of related conditions. Socioeconomic determinants^(9,10)^ are also associated with these factors, increasing CC incidence and mortality among the most vulnerable individuals^(11,12)^. The implementation of HPV vaccination processes represents the primary strategy for preventing infection in both men and women, and is a highly significant prevention strategy within public policy (PP)^(13,14)^.

To address inequalities, the Pan American Health Organization approved the 2018–2030 Action Plan, which aims to guarantee universal access to prevention, early detection, and vaccination measures, and to reduce the disease burden in order to eradicate this health problem in the long term^(1,15)^. Vaccination coverage in the Region of the Americas is expected to reach 80% in at least 15 countries^(15)^. However, this measure against HPV faces structural, social, and cultural barriers that limit equitable access to a high-level strategy^(16)^.

In Chile, the National Immunization Program, led by nursing professionals, guarantees universal and equitable access to all essential vaccines against vaccine-preventable diseases. Since the implementation of HPV vaccine in 2016, Exempt Decree 50 of 2021 has included a second dose for girls and boys starting at age 9^(17)^. Despite the mandatory nature of the program, vaccination coverage across the country has been uneven. While high rates were achieved between 2016 and 2019, a general decline in coverage was observed during 2020 and 2021—attributed to the COVID-19 pandemic—with rates falling to a low of 69.3% for the first dose and 56.1% for the second dose in 2021. In 2022, coverage rates reached 90.2% for the first dose, 63.6% for the second, and 91.8% for the complete regimen^(18)^. Although the Americas and Latin America have a long history of high vaccination coverage thanks to their efficient national immunization programs, the HPV vaccine uptake among the public has been lower than expected^(19)^. However, given their professional competence in healthcare, it is necessary to strengthen nursing professionals’ leadership in vaccination processes during the implementation of various health programs—both globally and nationally^(20)^—as they are uniquely positioned to promote HPV vaccination efforts, and their participation is a fundamental strategy for increasing vaccination coverage^(21,22)^.

The importance of assessing PPs is currently recognized, as it represents an opportunity for continuous improvement^(23)^and helps focus attention on all the components that influence their development. In the context of PP analysis, there is a need to employ robust conceptual models—notably the Walt and Gilson health policy triangle (HPT), developed in 1994. This model offers a simplified yet systematic approach to considering the holistic nature of PP development, focusing on factors that explain how and why policies change. These are interconnected elements that influence one another, enabling in-depth observations regarding who makes decisions (actors) and how (process), what decisions are made (content), and under what conditions (context)^(24,25,26)^.

Viewed in this light, this research aimed to examine and analyze the HPV vaccination policy implemented in Chile using the Walt and Gilson HPT framework—supported by a scoping review—with the aim of generating evidence regarding key actors, implementation dynamics, activities undertaken, and decisions made over time, thereby presenting a bottom-up framework^(27)^ to strengthen this policy and contribute to improved health outcomes.

## METHOD

### Study Design

This is a scoping review, structured according to JBI Evidence Synthesis Manual guidelines^(28)^, which allows for the analysis of emerging evidence, provides a comprehensive overview of a research question or knowledge gap, and guides the need for future research in these areas. The five stages recommended by JBI were adopted: 1) Research question identification; 2) Search for relevant studies; 3) Study selection; 4) Data mapping; 5) Grouping, summarizing, and presenting the results^(29,30)^.

This study drew upon the Walt and Gilson HPT. Content refers to ideas, policy objectives, sources of evidence, or a particular policy issue. Context refers to the social, economic, political, cultural, and environmental conditions that can affect PP. These contexts can be strongly influenced by public perception, ideologies, political instability, history, and cultural values and beliefs. The process refers to the policy process stages, including agenda-setting, formulation, implementation, and policy assessment. Actors represent individuals and organizations (national or regional governments, civil society groups, professional organizations, trade unions, or private-sector groups) that participate in or have an interest in a specific policy issue, as they act upon and influence it. The Walt and Gilson HPT connects these components, showing how they influence and interact with one another, while placing the actors at the center of the model^(23,24,26,30,31)^. The guiding question of this study was: how are the content, context, policy-making process, and actors comprising the Walt and Gilson HPT characterized within the HPV vaccination PP?

**Figure 1:**
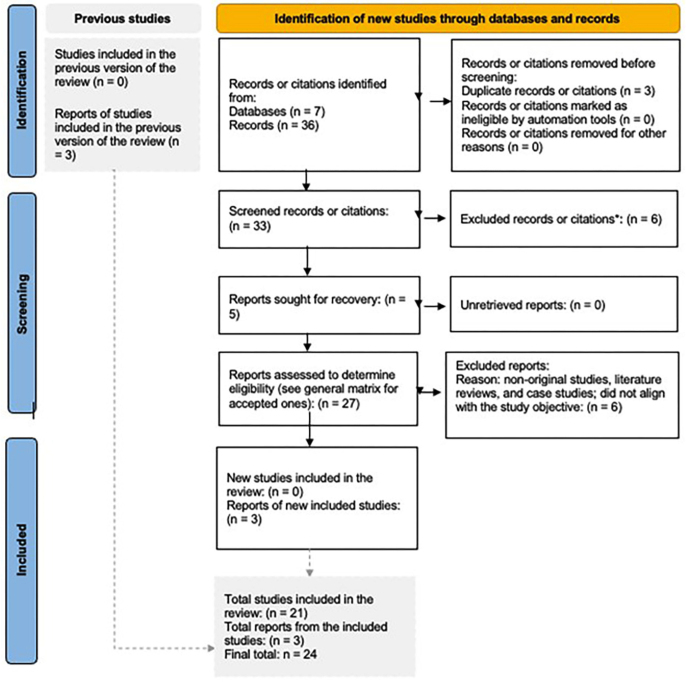
Preferred Reporting Items for Systematic reviews and Meta-Analyses extension for Scoping Reviews flowchart. Santiago, Chile, 2025.

Source: prepared by the author.

To prepare this report, the checklist based on Preferred Reporting Items for Systematic Reviews and Meta-Analyses extension for Scoping Reviews recommendations was used^(32)^see Figure 1.

### Selection Criteria

Qualitative and quantitative articles, critical analyses, case studies, and official websites and ministerial reports regarding the implementation of this PP in Chile were included. Unavailable online documents, texts relating to the pharmaceutical industry, therapies, research protocols, academic theses, monographs, editorials, expert opinions, abstracts, conference proceedings, and correspondence were excluded.

### Sources of Information

The search was conducted between July and September 2025 in the PubMed, Web of Science, CINAHL, Scopus, Google Scholar, and SciELO databases. The search period covers 2018 to 2024.

### Search Strategy

To verify the feasibility of the research, a broad, open preliminary search was conducted in the PubMed database. The following keywords, obtained from the DeCS/MeSH platform, were defined for this purpose: “Vaccines”; “Human Papillomavirus 31”; “Health Policy, Planning, and Management”; “Universal Health Care”; “Nursing Care”; “Health Policy”. To optimize results, the Boolean operators AND, OR, and NOT were applied, with search filters set for full-text availability (abstract and full text) and publication in Spanish, English, or Portuguese. Additionally, official documents were reviewed using a manual snowball search method. The database search strategy was as follows: PubMed -((“papillomavirus vaccines/administration and dosage” [MeSH Terms]) AND (policy making [MeSH Terms])) AND (“immunization programs” [MeSH Terms]); papillomavirus vaccines AND health policy AND Triangle; Web of Science - Papillomavirus Vaccines AND policy making; papillomavirus vaccines (All Fields) AND Health Policy (All Fields) AND triangle (All Fields); CINAHL - papillomavirus vaccine AND Health policy; Scopus - papillomavirus vaccines AND health policy AND Nursing; *Google Académico* - Papillomavirus vaccines AND triangle walt-gibson; Papillomavirus vaccines AND Policy making; Papillomavirus vaccines AND nursing; SciELO - papillomavirus humano 31 AND *enfermería*; *Papillomavirus humano 31* AND *formulación de políticas*.

### Selection Process

Text selection was organized by a team of researchers and validated by a third researcher with experience in this process, based on the research question and the study objectives. Duplicates were removed, and a critical review of titles and abstracts was conducted. Articles containing characteristics of the components of the Walt and Gilson HPT framework relevant to the study’s focus were included. Studies that did not address the primary research question were excluded. Study selection was based on the stated eligibility criteria.

### Data Processing

Data from the various studies were summarized using a narrative-qualitative synthesis^(33)^, which enabled the identification of characteristics within the Walt and Gilson HPT interactions. The studies comprising the final sample for the analysis were read in their entirety. Data collection was organized in a structured Microsoft Excel^®^ spreadsheet to facilitate qualitative-descriptive analysis, which included the following variables: title, year of publication, origin, language, design, databases, study objectives, design, and main results. Finally, the information was organized into a central matrix linked to the Walt and Gilson HPT components. No ethics committee is required.

## RESULTS

### Descriptive Analyses

Based on a structured literature search, 45% of studies were published between 2021 and 2022—predominantly in Asia (32%) and in English (100%)—employed quantitative research designs (45%), and were retrieved from PubMed (30%) (Table 1).

**Table 1:** Description of articles selected from a structured search (n = 21)– Santiago, Chile, 2025.

Category	Indicator	Frequency
Year of publication	Before 2018	2
	2019 – 2020	5
	2021 – 2022	10
	2023 – 2024	4
Geographic distribution	Asia	7
	Europe	4
	North America	3
	Central America	3
	Africa	2
	Middle East	2
Language	English	21
	Spanish	0
	Portuguese	0
Study design	Quantitative	9
	Qualitative	6
	Literature reviews	5
	Mixed methods	1
Database	PubMed	6
	Google Scholar	4
	Web of Science	4
	Manual search	2
	SciELO	2
	Scopus	2
	CINAHL	1

Source: prepared by the author.

Based on the literature review—comprising 21 articles obtained through a structured review, supplemented by three reports on policy implementation—it is evident that the components of the Walt and Gilson HPT have significantly influenced the implementation processes of HPV vaccination policy, both in Chile and elsewhere. However, challenges remain regarding health promotion and the prevention of HPV-related diseases (Chart 1).

**Chart 1:** Matrix of data obtained from a structured search (n = 21) – Santiago, Chile, 2025.

Id.	Title, authors, and year	Study objective	Key findings
1	The role of nurses as human papillomavirus vaccination advocates in China: perception from nursing students Lin *et al*., 2022^(34)^	Investigate nursing students’ perspectives regarding the role of nursing professionals in advocating for the Human Papillomavirus vaccine.	The majority of students expressed their intention to act as vaccination promoters and advisors; only one-third stated that they would not advocate for vaccination. The group was characterized by consisting of first-year students who were knowledgeable about the Human Papillomavirus, came from higher-income households, and had received the vaccine. The barriers identified were a lack of training and discomfort when discussing sexuality. Institutional support is key to strengthening the role of nursing professionals in promoting Human Papillomavirus vaccination.
2	Advancing Nursing Policy Advocacy Knowledge: A Theoretical Exploration Chiu, 2021^(35)^	Explore the knowledge and perspectives required by nurses to participate effectively in public policy.	The nursing profession must strengthen specific knowledge regarding the promotion of policies to achieve social justice. It must develop disciplinary knowledge based on conceptual and theoretical frameworks beyond nursing. Although nurses have always participated in public policy, the impact of their actions and the factors limiting their participation must be clearly understood.
3	Nurses’ and midwives’ knowledge, attitudes, and acceptance regarding human papillomavirus vaccination in Ghana: a cross-sectional study Ebu *et al*., 2021^(36)^	Assess the level of knowledge among nurses and midwives regarding risk factors for cervical cancer.	Fewer than half of participants were aware of the risk factors for cervical cancer and the Human Papillomavirus vaccine. Nearly half had undergone a Pap test, and vaccine acceptance was low. The barriers were lack of knowledge, cost, fear, and adverse effects. The facilitators were age, continuing education, and years of experience. It is recommended to strengthen training and reduce economic gaps.
4	Human papillomavirus vaccination 2020 guideline update: American Cancer Society (ACS) guideline adaptation Salow *et al*., 2020^(37)^	Adapt the current recommendations of the Advisory Committee on Immunization Practices for Human Papillomavirus vaccination.	Routine Human Papillomavirus vaccination between the ages of 9 and 12 is recommended to ensure timely vaccination. Healthcare professionals should offer vaccination starting at age 9 or 10. A booster dose should be administered to all individuals up to 26 years of age. Professionals must inform individuals aged 22 to 26 who have not been vaccinated. Booster Human Papillomavirus vaccination is not recommended for adults over the age of 26.
5	Drivers for human papillomavirus vaccination in Valencia (Spain) Navarro-Illana *et al*., 2018^(38)^	Describe the factors associated with Human Papillomavirus vaccination in adolescents and their parents’ opinions regarding the vaccine.	Human Papillomavirus vaccination coverage stands at 74.5%. Influencing factors include advice from healthcare personnel—particularly nursing staff— which has a significant impact on the other variables. The factors that most influence vaccination are the mother’s country of origin, the parents’ marital status, and the level of knowledge regarding the Human Papillomavirus. The variables favoring vaccination are the use of preventive services and the perception of risk. Education and training for the healthcare team must be strengthened.
6	An Integrative Review of the Influences on Decision-Making of Young People About Human Papillomavirus Vaccine Sisson y Wilkinson, 2019^(39)^	Explore and understand the factors influencing young people’s decisionmaking regarding Human Papillomavirus vaccination.	The decision to vaccinate is influenced by four factors: fear, pain, parental involvement, and recommendations from healthcare professionals. The last two are crucial for vaccination adherence, reinforcing the role of school nurses in promoting Human Papillomavirus vaccination. Strategies must be developed that incorporate social and cultural aspects to help build trust in healthcare personnel.
7	Barriers to and Facilitators of Human Papillomavirus Vaccination Among People Aged 9 to 26 Years: A Systematic Review Zheng *et al*., 2021^(40)^	Understand the barriers and facilitators faced by adolescents and young adults aged 9 to 26 in initiating Human Papillomavirus vaccination.	The barriers include a lack of knowledge about the virus and the vaccine, concerns regarding the risks of the procedure and vaccine efficacy, costs, fear of pain, past negative experiences, discrimination, and a lack of time. Recommendations from healthcare personnel serve as facilitators. Strengthening education and information campaigns regarding Human Papillomavirus vaccination can increase acceptance and improve vaccination rates.
8	Policy analysis of cervical cancer prevention in Iran based on the policy triangle model Pirani *et al*., 2023^(41)^	Analyze cervical cancer prevention policies in Iran based on Walt and Gilson’s health policy analysis triangle model.	The main problems with vaccination policy are the lack of oversight, assessment, and use of scientific evidence, as well as weaknesses in organizational structure, inter-sectoral coordination, and executive management. Deficiencies are also observed in the application of scientific principles, operational protocols, and the use of technologies. It is necessary to strengthen prevention, education, and professional training for public health decision-making.
9	The feasibility of universal HPV vaccination program in Shenzhen of China: a health policy analysis Chen y Wong, 2019^(42)^	Assess the feasibility of a universal Human Papillomavirus vaccination program for adolescent girls in order to identify strategies for policy formulation in Shenzhen, China.	There is growing interest in Human Papillomavirus vaccination, fostered by favorable living conditions in Shenzhen. Challenges persist regarding coordination among health institutions, the mobilization of policies, vaccination plans and strategies, and the commitment of key stakeholders such as parents and civil society organizations.
10	Human papillomavirus (HPV) vaccination in a privately funded program in Ghana: A qualitative case study Marfo *et al*., 2024^(43)^	Identify Human Papillomavirus vaccination processes, practice challenges, and policy interests.	The lack of a policy guiding the vaccination program affects its efficiency. The emphasis placed on sexual history and Human Papillomavirus screening as a basis for the vaccination decision is recognized as a limitation, compounded by the absence of formal Human Papillomavirus education programs for healthcare staff and vaccine recipients. Furthermore, there is a lack of support from leaders to drive these programs forward.
11	Implementing the free HPV vaccination for adolescent girls aged below 14 in Shenzhen, Guangdong Province of China: experience, challenges, and lessons Wu *et al*., 2023^(44)^	Assess a pilot project in Shenzhen, Guangdong Province, promote the inclusion of the Human Papillomavirus vaccine in the local immunization program and address existing barriers to its implementation.	The increase in vaccination was made possible by the government’s commitment, funding, collaboration among different sectors, and guaranteed access to this service. Challenges remain to be overcome, such as achieving universal coverage, addressing vaccine refusal by certain groups, and expanding the age range so that more people can access it. Success depends on the political will of states to strengthen public health policies.
12	Prevalence of high-risk human papillomavirus among women in two English-speaking Caribbean countries Andall-Brereton, 2017^(45)^	Characterize high-risk Human Papillomavirus infections in a sample of women from two small English-speaking Caribbean countries: Saint Kitts and Nevis and Saint Vincent and the Grenadines.	Twelve high-risk Human Papillomavirus genotypes were identified in English-speaking Caribbean countries, where the prevalence of this infection is high. Human Papillomavirus infections were associated with younger age, a higher frequency of abnormal cytology results, and higher parity. More than 20% of women presented with multiple infections, representing a higher cytological risk. These results support the implementation of the nonavalent vaccine in school-based programs.
13	Public health impact and cost effectiveness of routine and catch-up vaccination of girls and women with a nine-valent HPV vaccine in Japan: a model-based study Cody *et al*., 2021^(46)^	Assess the impact on population health and the cost-benefit ratio of routine vaccination of girls and women aged 11 to 26 years with quadrivalent or nonavalent Human Papillomavirus vaccines in Japan, compared with no vaccination.	The nonavalent vaccine is projected to have a major impact on public health in Japan, as it would reduce cervical cancer cases by more than 86%, thereby preventing over 450,000 new cases and more than 50,000 deaths. It would also have an impact on genital warts and other cancers related to the Human Papillomavirus. The nonavalent vaccine, with campaigns targeting adolescents, is effective in preventing cervical cancer. Vaccination programs should be expanded and made a public health priority.
14	Public health impact and costeffectiveness of a nine-valent gender-neutral HPV vaccination program in France Majed *et al*., 2021^(47)^	Assess the public health impact and cost-effectiveness of a gender-neutral vaccination program compared to a girls-only vaccination program.	Neutral vaccination achieved additional reductions in the incidence and mortality of Human Papillomavirus-related cancers. Other diseases related to the Human Papillomavirus were also prevented. A scenario with 60% coverage in both sexes significantly reduced the disease burden but increased the incremental cost-effectiveness ratio to €40.401. The inclusion of boys demonstrated public health benefits and supported expanding the vaccination program to include both sexes with the nonavalent vaccine.
15	Public health impact of 2-, 4-, and 9-valent HPV vaccination in females on cervical and noncervical diseases in men and women under different coverage scenarios in China: A simulation study Diakite *et al*., 2023^(48)^	Estimate the public health impact in China of Human Papillomavirus vaccination strategies (with and without cross-protection) using bivalent, quadrivalent, and nonavalent vaccines.	All vaccination strategies have a positive impact on reducing cervical cancer and other diseases associated with the Human Papillomavirus. The nonavalent vaccine demonstrates greater effectiveness, projecting a reduction of up to 5 million cases over 100 years. It has been demonstrated that primary vaccination with any type of Human Papillomavirus vaccine is the primary factor in reducing the burden of this disease.
16	Toward universal human papillomavirus vaccination for adolescent girls in Hong Kong: a policy analysis Chen *et al*., 2020^(49)^	Examine stakeholder participation and interactions in promoting a universal Human Papillomavirus vaccination program, using Walt and Gilson’s health policy analysis triangle framework for structured health policy analysis.	The political system, together with the values inherent to the culture and the influence of the West, shapes how people perceive the development of Human Papillomavirus vaccination policies. Identify diverse ethical stances and an uneven use of scientific evidence. To advance vaccination efforts, universal and free access for adolescents must be guaranteed, while also reinforcing strategies that promote social acceptance of this type of procedure.
17	Content analysis of digital media coverage of the human papillomavirus vaccine schoolentry requirement policy in Puerto Rico Colón *et al*., 2021^(50)^	Analyze the content of digital news coverage regarding the implementation of the Human Papillomavirus vaccination policy in Puerto Rico.	Most media outlets did not mention the number of doses, and 79% highlighted it as a strategy for preventing cervical cancer. They presented neutral content regarding the vaccine and cancers associated with the Human Papillomavirus. The negative messages included information about political autonomy. The positive aspects were related to sex education and cancer prevention. Limited and ambivalent communication can affect the acceptance and coverage of the Human Papillomavirus vaccine.
18	Awareness of human papillomavirus and acceptability of the vaccine among women in Palestine: is it time for policy adjustment? Elshami *et al*., 2022^(51)^	Assess the level of knowledge among Palestinian women regarding the Human Papillomavirus, as well as the level of acceptance and knowledge concerning the vaccine and the factors associated with them.	Knowledge regarding the Human Papillomavirus and its vaccine was extremely low. Only 8.1% of women had heard of the Human Papillomavirus, and only 46 women in this group knew about the vaccine. The majority expressed a willingness to be vaccinated or have their daughters vaccinated, provided the vaccine was free or had an affordable copayment. Incorporating the Human Papillomavirus vaccine into a national program can improve acceptance and awareness.
19	Health policy analysis of the non-implementation of HPV vaccination coverage in the pay for performance scheme in France Ohannessian *et al*., 2019^(52)^	Analyze the reasons for the non-implementation of an indicator for the Human Papillomavirus vaccine in France’s National Cancer Control Plan.	The public policy actors were the Ministry of Health and the French Cancer Institute, which formulated it; public insurance, which was its indirect objective, and the doctors, who carried it out directly. The public policy lacked evidence-based foundations and was not taken into account in the National Cancer Control Plan report. The context was marked by vaccine resistance and ethical concerns within primary care, in opposition to the Ministry of Health. The process involved multiple stakeholders and complex governance. These conditions may have contributed to the failure of the public policy on Human Papillomavirus vaccination in France.
20	Implementation of the human papillomavirus school-entry requirement in Puerto Rico: barriers and facilitators using the consolidated framework for implementation research Colón-López *et al*., 2021^(53)^	Identify barriers and facilitators for the implementation of the Human Papillomavirus vaccine among students aged 11 to 12 in Puerto Rico, using the Consolidated Framework for Implementation Research.	The facilitator must be aware of the needs of parents and students regarding meeting vaccination requirements prior to school entry. Parents supported the implementation of public policy initiatives that would back other vaccination strategies. The barriers were the cost of vaccine administration by private providers, the influence of social media, and the shortage of school nurses as key personnel.
21	Evolution of Public Health Human Papillomavirus Immunization Programs in Canada Goyette *et al*., 2021^(54)^	Describe the evolution of these vaccination programs in Canada.	Vaccination of school-age girls began during the 2007/08 to 2010/11 school years with three doses of the quadrivalent Human Papillomavirus vaccine in all provinces, except Quebec. Most provinces include catch-up vaccination and vaccination for high-risk groups. Vaccination rates vary markedly among provinces; only in Prince Edward Island do they exceed 80%.

Source: prepared by the author.

### Core Components of the Walt and Gilson Health Policy Triangle

As frontline healthcare actors, healthcare professionals in general—and nursing professionals in particular—are uniquely positioned to increase public acceptance of vaccination processes, coverage, and access. Concerning content, context, and process, new strategies are needed to broaden acceptance of the process—given the presence of dynamic environments—and to formulate effective, evidence-based vaccination policies (Chart 2).

**Chart 2:** Components of the Walt and Gilson health policy triangle, authors, and distinctive theoretical elements comprising the articles obtained in the structured review and reports related to policy implementation – Santiago, Chile, 2025.

Component	Authors	Distinctive elementsStudy objective
Actors	Lin *et al.*, 2022^(34)^	Healthcare professionals play a leading role in Human Papillomavirus vaccination processes. However, nursing professionals are in a privileged position to foster public acceptance of the Human Papillomavirus vaccine, acting as key players in increasing coverage and access. In this regard, national and international bodies exert influence on the implementation processes of this public policy.
	Chiu, 2021^(35)^	
	Ebu *et al.*, 2021^(36)^	
	Salow *et al.*, 2020^(37)^	
	Navarro *et al.*, 2018^(38)^	
	Sisson *et al.*, 2019^(39)^	
	Zheng *et al.*, 2021^(40)^	
	Pirani *et al.*, 2023^(41)^	
Content	Chen *et al.*, 2019^(42)^	An effective Human Papillomavirus vaccination policy—and the promotion of universal, free Human Papillomavirus vaccination—requires new strategies to increase acceptance of the process, formulate policies based on facts and values, link research with policymaking, and improve the formation of publicprivate coalitions.
	Marfo *et al.*, 2024^(43)^	
	Wu *et al.*, 2023^(44)^	
	Pirani *et al.*, 2023^(41)^	
	Andall-Brereton *et al.*, 2017^(45)^	
	Cody *et al.*, 2021^(46)^	
	Majed *et al.*, 2021^(47)^	
	Diakite *et al.*, 2023^(48)^	
Context	Chen *et al.*, 2019^(42)^	Despite advances, cervical cancer continues to affect people living in diverse contexts and dynamic environments. It is recommended to fund prevention programs, raise public awareness, provide education on self-care, and adjust university training, as these are key strategies for strengthening detection and supporting evidence-based public health policy decision-making.
	Marfo *et al.*, 2024^(43)^	
	Wu *et al.*, 2023^(44)^	
	Chen *et al.*, 2020^(49)^	
	Colón *et al.*, 2021^(50)^	
	Elshami *et al.*, 2022^(51)^	
	Ohannessian *et al.*, 2019^(52)^	
	Navarro *et al.*, 2018^(38)^	
	Colón *et al.*, 2021^(53)^	
	Zheng *et al.*, 2021^(40)^	
	Pirani *et al.*, 2023^(41)^	
	Andall-Brereton *et al.*, 2017^(45)^	
	Sisson *et al.*, 2019^(39)^	
Process	Chen *et al.*, 2019^(42)^	Since their introduction, Human Papillomavirus vaccination programs in Chile and worldwide have seen their schedules and dosages evolve. In this regard, it is necessary to face the challenges of achieving universal coverage, which include addressing vaccine hesitancy, expanding the program to cover a wider age range, and ensuring the consistent quality of vaccination services within primary health care. Overcoming these challenges will require innovative strategies, public-private partnerships, funding, sustainable strategies, and the development of standardized guidelines to support evidence-based Human Papillomavirus vaccination practice.
	Marfo *et al.*, 2024^(43)^	
	Wu *et al.*, 2023^(44)^	
	Pirani *et al.*, 2023^(41)^	
	Goyette *et al.*, 2021^(54)^	
	Elshami *et al.*, 2022^(51)^	
	Ohannessian *et al.*, 2019^(52)^	
	Colón *et al.*, 2021^(53)^	
	Technical-Operational Guidelines for School-Based Vaccination 2025^(55)^	
	Ord. 988: report on Human Papillomavirus vaccination catch-up, Chile, 2025^(56)^	
	Order 1032 (B27N°): Human Papillomavirus vaccine update reports for 5th-grade students, Chile, 2025^(57)^	

Source: Prepared by the authors.

## DISCUSSION

Conducting our research process enabled us to examine and analyze the HPV vaccination policy implemented in Chile through the lens of the Walt and Gilson HPT framework and to identify areas for improvement, while consistently focusing on the policy’s content, contexts, processes, and actors—the elements situated at the core of the model. This bottom-up analysis approach provided a broader view of the PP, as it allows for the analysis of its specific details and components.

### Actors

In the Walt and Gilson HPT model, actors are identified as individuals and organizations that act upon and influence a PP. In HPV vaccination policy, both vaccine recipients and those who provide the clinical procedure play a fundamental role. This preventive measure is more than a mere technical decision adopted by a scientific committee and health departments, implemented by professionals, and received by the public—it represents a key public health strategy to address the incidence of one of the leading causes of death among women: HPV-associated CC^(58)^.

Lin et al.^(34)^ maintain that nursing professionals play a leading role in vaccination and are uniquely positioned to foster acceptance of HPV vaccine, making them key players in increasing vaccination coverage. These frontline professionals implement and restructure vaccination programs, which has a fundamental impact on health outcomes^(59)^. Sisson and Wilkinson^(39)^ stated that nursing professionals must consider that young people’s decisions regarding HPV vaccination are influenced by multiple factors, including fear, risk perception, pain, parental involvement, and the influence of healthcare professionals themselves. However, precisely to strengthen healthcare coverage and social justice, Chiu’s 2021 study^(35)^ asserts the importance of the nursing profession developing knowledge and political leadership to strengthen healthcare systems and global priorities. As for the improvement of HPV vaccination processes, Lin et al.^(34)^, Ebu et al.^(36)^, Salow et al.^(37)^, and Navarro-Illana et al.^(38)^ agree that, on the one hand, institutional support is needed to develop health education programs for nursing professionals—acting as promoters of HPV vaccine—and, on the other, knowledge and attitudes regarding HPV must be strengthened, and the stigma associated with discussing HPV-related topics with individuals must be eliminated.

Furthermore, among those receiving this clinical procedure, recommendations from healthcare providers serve as important facilitators, acting as a key driver in the decision to be vaccinated for both adolescents and young adults^(34)^. In this regard, Navarro-Illana et al.^(38)^, Sisson and Wilkinson^(39)^, Zheng et al.^(40)^, and Pirani et al.^(41)^ emphasize that—in the face of barriers to HPV vaccination perceived by the public, which primarily include a lack of knowledge, concerns regarding vaccine safety and efficacy, cost-related issues, fear of pain, previous negative experiences with vaccines, discrimination, and a lack of time—education and campaigns aimed at increasing awareness of HPV susceptibility and transmission, as well as clarifying the vaccine’s safety and effectiveness, are strategies that can promote acceptance and increase vaccination rates.

### Content

In the Walt and Gilson HPT, the operational aspect concerning content encompasses policy objectives, guidelines, regulations, concrete actions, and directives that facilitate the implementation process. In this context, this institutional “what to do” mobilizes resources and specific strategies capable of strengthening the health outcomes being pursued. Thus, the effectiveness of the HPV vaccination policy, health promotion, and the prevention of life-threatening diseases requires new strategies to broaden acceptance of the process, formulate policies based on facts and collective values, link research with policymaking, and improve the formation of public-private coalitions. In this regard, the 2019 study by Chen and Wong^(42)^ concludes that, to achieve higher standards of CC prevention, systematic HPV vaccination processes must be implemented for individuals aged 9 to 12, alongside HPV booster doses for all individuals up to age 26 who have not been adequately vaccinated. For individuals over the age of 26, HPV booster vaccination is not recommended, as it represents a less effective public health strategy for reducing the risk of CC.

According to Marfo et al.^(43)^, although many vaccinators advocate for a universal HPV vaccination program, some policymakers and program managers are reluctant to prioritize its promotion. This creates limitations for preventive strategies, as a vaccination program lacking a formal policy may be constrained in terms of quality and efficiency, given the absence of accountability measures and sustainability. This is where standardized guidelines and protocols are needed to support evidence-based HPV vaccination practice. Pirani et al.^(41)^ also emphasize the need to strengthen PPs that support these health processes, complementing this with the need to fund prevention programs, raise public awareness, provide self-care education, and adjust university training, as these represent key strategies for enhancing detection and supporting decision-making within public health policies.

In terms of the strengthening of health processes to invigorate the HPV vaccination strategy, Wu et al.^(44)^ in 2023 not only address vaccination hesitancy through public awareness, education and health communication actions about HPV vaccine from primary care, but also emphasize that, to meet the challenges of universal vaccination coverage, a firm government commitment and political will, sufficient funding, multisectoral public-private collaboration, and guaranteed accessibility are required and vaccine affordability. On the other hand, Andall-Brereton et al.^(45)^, Cody et al.^(46)^, Majed et al.^(47)^, and Diakite et al.^(48)^ provide further key evidence regarding HPV prevalence and risk factors, asserting the need to move away from the bivalent vaccine and urgently incorporate the nonavalent vaccine into HPV immunization programs—leveraging school-based healthcare systems—as this will prevent a significant number of cases of HPV-related diseases in both men and women.

### Context

Despite advances in HPV vaccination coverage, social, economic, political, cultural, and environmental conditions affect the target population, causing related CC to continue affecting people in all regions of the world. In this regard, the literature emphatically points out that raising public awareness, educating on self-care, and adjusting university training—so that new healthcare professionals can influence people in these areas—are key strategies for strengthening health promotion and the prevention of associated diseases, enhancing detection, and supporting the implementation of local health programs.

According to Chen and Wong^(42)^ (2019) and Marfo et al.^(43)^, the quality and efficiency of HPV vaccination programs require a deep understanding of the characteristics of the target groups for this health measure, as this enables evidence-based support for HPV vaccination policies. Complementing this, Wu et al.^(44)^and Chen et al.^(49)^ (2020) assert that the sustainability of HPV vaccination will depend on the political will to incorporate preventive interventions—grounded in both facts and values—into policy, while taking into account contextual factors that may influence their implementation in different settings.

Communication strategies and the information available to communities are also a cause for concern. According to Colón-López et al.^(50)^, neutral and negative topics regarding HPV could influence public concern regarding HPV vaccination rates. On the other hand, Elshami et al.^(51)^ discussed how the low level of general knowledge regarding HPV affects vaccination against this virus. Both situations can influence the governance of vaccination policies and contribute to creating unfavorable contexts for policy, according to Ohannessian et al.^(52)^. However, according to Navarro-Illana et al.^(38)^, Sisson and Wilkinson^(39)^, Colón et al.^(50)^ (in 2021), Zheng et al.^(40)^, and Pirani et al.^(41)^, most barriers to this policy can be overcome through the implementation of school-based education and training programs, public awareness-raising, and self-care education—measures that can support this health policy, as emphasized by Andall-Brereton et al.^(45)^, while facilitating its acceptance and improving HPV immunization rates.

### Process

Since their introduction, HPV vaccination programs have evolved in terms of schedules and dosages to address coverage challenges—amidst territorial and social diversity—through various strategies for the development and implementation of this public health policy. The literature analyzed emphasizes that overcoming these challenges will require evidence-based HPV vaccination strategies that identify age groups for whom vaccination yields the highest indicators of efficacy and health efficiency regarding the prevention of associated diseases, as suggested by Chen et al. (2019)^(42)^, Marfo et al.^(43)^, Wu et al.^(44)^, and Goyette et al.^(54)^.

Regarding assessment, adaptations, modifications, and adjustments must be made to the implementation process of this PP. Colón et al.^(50)^, Pirani et al.^(41)^, Elshami et al.^(51)^, and Ohannessian et al.^(52)^ state that strengthening civic education programs—specifically by raising public awareness regarding these matters—could ensure sustainable strategies for the coverage, access, and timeliness of this health initiative.

In this context, one of the major challenges for HPV vaccination programs is to implement strategies that ensure the long-term sustainability of the public health program, achieving target coverage levels among the target populations. Vaccination policies in Chile serve as an example of this, as they are guaranteed by law through the nonavalent HPV vaccine^(17)^. Ensuring this requires strategies such as bringing healthcare professionals closer to the community—through campaigns in schools^(55)^ or by reaching out to underserved groups^(56,57)^—in order to achieve the target coverage. The success of an initiative as momentous as the prevention and eventual eradication of CC requires the commitment of the healthcare team and sustained support from the State to ensure equity and timeliness for the benefit of the population.

The limitations identified in this study relate to the difficulty of accessing official assessment reports on the implementation processes of this policy within the national territory. Similarly, the scarcity of studies in Latin America poses challenges for research teams and governments as they seek to transition toward comprehensive and sustainable human development.

## CONCLUSION

This study demonstrated that the analysis of PPs—facilitated by the Walt and Gilson HPT framework—enables a comprehensive characterization of health policy, as it identifies the interactions between context, content, and process, and reveals how the actors serve as key protagonists within the model. From a bottom-up perspective, each component of the Walt and Gilson HPT demonstrates that PP is dynamic and requires continuous adaptation to social realities and people’s needs. While HPV vaccination represents one of the safest and most effective interventions against virus-related cancers—both in Chile and in other communities—the role of healthcare professionals in general, and nursing professionals in particular, demands active participation in all PP cycle phases, from design and decision-making to implementation and assessment. To this end, its leadership capacity, its close connection to the community—forged through the strategic link between the population’s needs and the guarantees provided by the State—and its holistic vision of patient care position this discipline as a key agent in promoting strategies such as HPV vaccination, thereby ensuring that the right to health is realized for the most vulnerable groups. This is a global priority that aims for comprehensive and sustainable human development.

## Data Availability

The entire dataset supporting the results of this study was published in the article itself.
