# Supplementary material for: Public policy on Human Papillomavirus immunization: an analysis based on the Walt and Gilson policy triangle
Source: Rev Esc Enferm USP. 2026 Aug 14;60:e20250521. doi: 10.1590/1980-220X-REEUSP-2025-0521en (PMC13475799; doi:10.1590/1980-220X-REEUSP-2025-0521en)
Supplement: Translation

# Translation: Política pública de inmunización contra Virus del Papiloma Humano: análisis desde el triángulo de Walt y Gibson

**DOI:** 10.1590/1980-220X-REEUSP-2025-0521es

**Authors:** María Angélica Saldías-Fernández, Jéssica García-Gutiérrez

**Affiliations:** 1 Universidad de Chile, Facultad de Medicina, Departamento de Enfermería, Santiago, Chile.

**Keywords:** Vacunas, Papillomavirus Humano 31, Políticas, Planificación y Administración en Salud, Atención de Salud Universal, Atención de Enfermería, Política de Salud

## Abstract

**Objetivo:**

Examinar y analizar la política pública de vacunación contra el Virus del Papiloma Humano implementada en Chile desde la perspectiva del triángulo de análisis de políticas de salud de Walt y Gilson.

**Método:**

Revisión de alcance, estructurada según las directrices del Manual de Síntesis de Evidencia del JBI para identificar las áreas generales de: actores, contenido, contexto y proceso.

**Resultados:**

De 24 artículos e informes oficiales seleccionados, los profesionales de enfermería son actores en una posición privilegiada para aumentar la aceptación ciudadana, cobertura y acceso a la vacunación. Respecto a contenido, contexto y proceso, se necesitan nuevas estrategias para ampliar la aceptación del proceso dada la presencia de entornos dinámicos y formular políticas de vacunación eficaces, basadas en la evidencia.

**Conclusión:**

El análisis de políticas públicas, mediado por el triángulo de Walt y Gilson, permite identificar las interacciones entre sus componentes y clarificar los desafíos relacionados con la promoción y prevención de enfermedades asociadas al Virus del Papiloma Humano, contribuyendo al fortalecimiento de la salud colectiva, los sistemas de salud y las prioridades globales orientadas al desarrollo humano integral y sostenible.

## INTRODUCCIÓN

El cáncer cervicouterino (CaCu) constituye un problema de salud pública en los países de ingresos bajos y medios^(1,2)^, concentrándose en regiones como Asia, África y Latinoamérica^(3)^. En los países de altos ingresos, la incidencia y mortalidad han disminuido en los últimos 20 años, asociándose con estrategias preventivas y de pesquisa temprana de casos^(4)^. En Chile, este tipo de cáncer es el tercero más común entre las mujeres, y afecta directamente a las etapas productivas y reproductivas^(4)^. Durante el año 2022, fallecieron 646 mujeres a causa del CaCu, lo cual se tradujo en una tasa de mortalidad ajustada de 6,07 por 100.000 habitantes^(5)^.

Uno de los principales factores etiológicos del CaCu es el Virus del Papiloma Humano (VPH)^(6)^, con capacidad fisiopatológica para generar una infección asintomática de largo plazo, que interfiere en la proliferación y diferenciación celular del cuello del útero, ocasionando lesiones de características neoplásicas e invasivas^(7)^. La literatura señala que el desarrollo del CaCu responde a factores sociales y contextuales^(8)^ que se manifiestan en forma de profundas inequidades en el acceso a mecanismos de prevención, así como en el tamizaje, diagnóstico y tratamiento oportunos de las patologías relacionadas. A estos factores se asocian además determinantes socioeconómicos^(9,10)^que elevan la incidencia y la mortalidad de la enfermedad en las personas más vulnerables^(11,12)^. La implementación de procesos de vacunación contra el VPH representa la principal estrategia de prevención primaria de la infección, tanto en hombres como en mujeres, y es una estrategia de prevención de gran relevancia como política pública (PP)^(13,14)^.

Haciendo frente a las desigualdades, en el 2018 la Organización Panamericana de la Salud aprobó el Plan de Acción 2018–2030, cuyo objetivo es garantizar el acceso universal a las medidas de prevención, detección precoz y vacunación, y lograr un impacto en la reducción de la carga de la enfermedad para, a largo plazo, erradicar este problema de salud^(1,15)^. Se espera que la cobertura de vacunación en la región de las Américas alcance un 80% en al menos 15 países^(15)^. Sin embargo, esta medida contra el VPH enfrenta barreras estructurales, sociales y culturales que limitan el acceso equitativo a una estrategia de alto nivel^(16)^.

En Chile, el Programa Nacional de Inmunizaciones, liderado por profesionales de enfermería, garantiza el acceso universal y equitativo a todas las vacunas esenciales contra enfermedades inmunoprevenibles. Desde la implementación de vacuna VPH en 2016, el Decreto Exento N°50 de 2021 incluyó la segunda dosis a partir de los 9 años para niñas y niños^(17)^. A pesar de la obligatoriedad, el país presenta coberturas dispares, ya que, si bien entre 2016 y 2019 se alcanzaron altas tasas de vacunación, durante los años 2020 y 2021 se observó una disminución de las coberturas de vacunación en general, atribuida a la situación de pandemia de COVID-19, llegando en 2021 a un mínimo del 69,3% y del 56,1% con la primera y la segunda dosis, respectivamente. En 2022, las coberturas alcanzaron 90,2% con la primera dosis, 63,6%, con la segunda, y 91,8%, con el esquema completo^(18)^. Aunque América y América Latina tienen una larga historia de alta cobertura de vacunación gracias a sus eficientes programas nacionales de inmunización, la adopción de la vacuna contra el VPH ha sido inferior a la esperada entre la ciudadanía^(19)^. Sin embargo, dada su competencia profesional en el cuidado de la salud de las personas, es necesario fortalecer el liderazgo de los profesionales de enfermería en los procesos de vacunación en la implementación de diversas PPs de salud, tanto a nivel mundial como nacional^(20)^, ya que se encuentran en una posición privilegiada para promover los procesos de vacunación contra el VPH, y su participación es la estrategia fundamental para aumentar la cobertura de vacunación^(21,22)^.

En la actualidad, es reconocida la importancia de evaluar PPs, pues representa una oportunidad de mejora continua^(23)^y contribuye a poner atención a todos los componentes que influyen en su desarrollo. Frente al análisis PP, se plantea la necesidad de usar modelos conceptuales sólidos, destacando entre ellos el triángulo de Walt y Gilson (TWG), diseñado en 1994, que ofrece un enfoque simplificado pero sistemático para considerar la naturaleza holística del desarrollo de PPs, prestando atención a factores que explican cómo y por qué cambian las políticas. Se trata de elementos interconectados que influyen entre sí y que permiten realizar observaciones profundas respecto a quién (actores) y cómo (proceso) se toman las decisiones, qué decisiones (contenido) se toman y bajo qué condiciones (contexto)^(24,25,26)^.

Mirado así, el objetivo de la presente investigación es examinar y analizar la política de vacunación contra el VPH implementada en Chile desde la perspectiva TWG y apoyada por revisión de alcance, con el propósito de generar evidencia sobre los actores relevantes, las dinámicas de implementación, las actividades realizadas y las decisiones tomadas a lo largo del tiempo, y así presentar un marco de referencia tipo *bottom-up* ^(27)^para fortalecer esta política y contribuir a mejorar los resultados en salud.

## MÉTODO

### Tipo de Estudio

Revisión de alcance, estructurada según las directrices del Manual de Síntesis de Evidencia del JBI^(28)^, que permite analizar la evidencia emergente, proporcionar una visión general completa de una pregunta de investigación o vacío de conocimiento y orientar la necesidad de futuras investigaciones en estas materias. Se adoptaron las cinco etapas recomendadas por JBI: 1) Identificación de la pregunta de investigación; 2) Búsqueda de estudios relevantes; 3) Selección de estudios; 4) Mapeo de datos; 5) Agrupación, resumen y presentación de los resultados^(29,30)^.

Este estudio se apoyó en el TWG. El contenido se refiere a las ideas, los objetivos de las políticas, las fuentes de evidencia o un asunto político en particular. El contexto se refiere a las condiciones sociales, económicas, políticas, culturales y ambientales que pueden afectar a la PP. Estos contextos pueden verse fuertemente influenciados por la percepción pública, las ideologías, la inestabilidad política, la historia, los valores y creencias culturales. El proceso se refiere a las etapas del proceso político, incluyendo el establecimiento de la agenda, la formulación, la implementación y la evaluación de las políticas. Los actores representan individuos y organizaciones (gobiernos nacionales o regionales, grupos de la sociedad civil, organizaciones profesionales, sindicatos o grupos del sector privado) que participan o tienen interés en un tema político específico, pues actúan e influyen en ello. El TWG conecta estos componentes, mostrando cómo influyen e interactúan entre sí, ubicando a los actores en el centro del modelo^(23,24,26,30,31)^. La pregunta orientadora de este estudio fue: ¿Cómo se caracterizan el contenido, contexto, proceso de formulación de políticas y actores que componen TWG en la PP de vacunación contra el VPH?

Para la elaboración de este informe, se utilizó la lista de verificación de acuerdo con las recomendaciones de la extensión para revisiones exploratorias de la Declaración *Preferred Reporting Items for Systematic reviews and Meta-Analyses extension for Scoping Reviews* ^(32)^ revisar Figura 1.

**Figura 1:** Diagrama de flujo Preferred Reporting Items for Systematic reviews and Meta-Analyses extension for Scoping Reviews. Santiago, Chile, 2025.

Fuente: elaboración propia.

### Criterios de Selección

Se incluyeron artículos cualitativos y cuantitativos, análisis críticos y estudios de caso, así como sitios web oficiales e informes ministeriales relativos a la implementación de esta PP en Chile. Se excluyeron documentos en línea no disponibles, textos relativos a la industria farmacéutica, terapias, protocolos de investigación, tesis académicas, monografías, artículos editoriales, opiniones de expertos, resúmenes, actas de congresos y correspondencia.

### Fuentes de Información

La búsqueda se realizó entre julio y septiembre de 2025 en las bases de datos PubMed, *Web of Science*, CINAHL, Scopus, Google Académico y SciELO. El período de búsqueda abarca de 2018 a 2024.

### Estrategia de Búsqueda

Para verificar la viabilidad de la investigación, se realizó una búsqueda preliminar amplia y abierta en la base de datos PubMed. En ella se definieron las siguientes palabras clave obtenidas desde la plataforma DeCS/MeSH: “Vacunas”; “Papillomavirus Humano 31”; “Políticas, Planificación y Administración en Salud”; “Atención de Salud Universal”; “Atención de Enfermería”; “Política de Salud”. Para optimizar los resultados, se aplicaron los operadores booleanos *AND*, *OR* y *NOT*, estableciendo como filtros de búsqueda la disponibilidad de texto completo (*abstract* y *full text*) y la publicación en idiomas español, inglés o portugués. En adición, se revisaron documentos oficiales, mediante método de búsqueda manual de bola de nieve. La estrategia de búsqueda en las bases de datos fue la siguiente: PubMed - ((“*papillomavirus vaccines/administration and dosage*” [*MeSH Terms*]) *AND* (*policy making* [*MeSH Terms*])) *AND* (“*immunization programs*” [*MeSH Terms*]); *papillomavirus vaccines AND health policy AND Triangle*; *Web of Science* - *Papillomavirus Vaccines AND policy making; papillomavirus vaccines* (*All Fields*) *AND Health Policy* (*All Fields*) *AND triangle* (*All Fields*); CINAHL - *papillomavirus vaccine AND Health policy*; Scopus - *papillomavirus vaccines AND health policy AND Nursing*; Google Académico - *Papillomavirus vaccines AND triangle walt-gibson; Papillomavirus vaccines AND Policy making; Papillomavirus vaccines AND nursing*; SciELO - papillomavirus humano 31 *AND* enfermería; Papillomavirus humano 31 *AND* formulación de políticas.

### Proceso de Selección

La selección de textos fue organizada por un equipo de investigadores y validada por un tercer investigador con experiencia en este proceso, con base en la pregunta de investigación y los objetivos de estudio. Se eliminaron duplicados, y se realizó una lectura crítica de los títulos y resúmenes. Se incluyeron artículos que contenían características de los componentes del TWG asociados con el enfoque del estudio. Se excluyeron los estudios que no abordaban la pregunta de investigación principal. La selección de los estudios se basó en los criterios de elegibilidad declarados.

### Procesamiento de Datos

Los datos de los diferentes estudios fueron resumidos mediante una síntesis de tipo narrativa-cualitativa^(33)^, que permitieron identificar las características en interacciones de TWG. Los estudios que conformaron la muestra final para el análisis se leyeron en su totalidad. La recopilación de datos se organizó en una hoja de cálculo estructurada de *Microsoft Excel* ^®^ para facilitar el análisis cualitativo-descriptivo, que incluyó las siguientes variables: título, año de publicación, origen, idioma, diseño, bases de datos, objetivos de estudio, diseño y resultados principales. Finalmente, se organizó la información en una matriz central relacionada con los componentes TWG. No se requiere comité de ética.

## RESULTADOS

### Análisis Descriptivos

A partir de la búsqueda bibliográfica estructurada de la literatura, un 45% fueron publicados entre 2021-2022, mayoritariamente en Asia (32%) y en idioma inglés (100%), aplicando metodologías de diseño cuantitativo (45%) y extraídos desde PubMed (30%) (Tabla 1).

**Tabla 1:** Descripción de artículos seleccionados a partir de búsqueda estructurada (n = 21) – Santiago, Chile, 2025.

Categoría	Indicador	Frecuencia
Año de publicación	Antes de 2018	2
	2019 – 2020	5
	2021 – 2022	10
	2023 – 2024	4
Distribución geográfica	Asia	7
	Europa	4
	Norteamérica	3
	Centroamérica	3
	África	2
	Medio Oriente	2
Idioma	Inglés	21
	Español	0
	Portugués	0
Diseño de estudio	Cuantitativo	9
	Cualitativo	6
	Revisiones de literatura	5
	Métodos mixtos	1
Base de datos	PubMed	6
	Google Académico	4
	*Web of Science*	4
	Búsqueda manual	2
	SciELO	2
	Scopus	2
	CINAHL	1

Fuente: elaboración propia.

A partir de la revisión bibliográfica, compuesta por 21 artículos obtenidos a partir de la revisión estructurada, a los que se suman tres informes relacionados con la aplicación de la política, se desprende que los componentes del TWG han influido notablemente en los procesos de aplicación de la política de vacunación contra el VPH, tanto en Chile como en otros lugares. No obstante, se identifican desafíos en materia de promoción de la salud y prevención de enfermedades asociadas al VPH (Cuadro 1).

**Cuadro 1:** Matriz de datos obtenidos a partir de búsqueda estructurada (n = 21) – Santiago, Chile, 2025.

Id.	Título, autores y año	Objetivo del estudio	Hallazgos principales
1	*The role of nurses as human papillomavirus vaccination advocates in China: perception from nursing students* Lin *et al.*, 2022^(34)^	Investigar las perspectivas de los estudiantes de enfermería con respecto al papel de los profesionales de enfermería en la defensa de la vacuna contra el Virus del Papiloma Humano.	La mayoría de los estudiantes manifestaron su intención de ser promotores de la vacunación y consejeros; solo un tercio declaró que no abogaría por la vacunación. El grupo se caracterizó por estar compuesto por estudiantes de primer año, tener conocimientos sobre el Virus Del Papiloma Humano, proceder de hogares con mayor nivel de ingresos y haber recibido la vacuna. Las barreras identificadas fueron la falta de capacitación y la incomodidad a la hora de hablar de sexualidad. El apoyo institucional es clave para fortalecer el papel del profesional de enfermería como promotor de la vacunación contra el Virus del Papiloma Humano.
2	*Advancing Nursing Policy Advocacy Knowledge: A Theoretical Exploration* Chiu, 2021^(35)^	Explorar los conocimientos y perspectivas que requieren las enfermeras para participar eficazmente en las políticas públicas.	La enfermería debe fortalecer el conocimiento específico en la promoción de políticas para lograr la justicia social. Debe desarrollar conocimiento disciplinar basado en marcos conceptuales y teóricos más allá de la enfermería. Aunque las enfermeras siempre han participado en la política pública, debe quedar claro el impacto de sus acciones y los factores que limitan su participación.
3	*Nurses’ and midwives’ knowledge, attitudes, and acceptance regarding human papillomavirus vaccination in Ghana: a cross-sectional study* Ebu *et al.*, 2021^(36)^	Evaluar el nivel de conocimiento de las enfermeras y parteras sobre los factores de riesgo del cáncer cervicouterino.	Menos de la mitad de las participantes conocían los factores de riesgo del cáncer cervicouterino y la vacuna contra el Virus del Papiloma Humano. Cerca de la mitad se había realizado la prueba del Papanicolau y la aceptación de la vacuna fue baja. Las barreras fueron el desconocimiento, el coste, el miedo y los efectos adversos. Los facilitadores fueron la edad, la educación continua y los años de experiencia. Se recomienda fortalecer la formación y reducir las brechas económicas.
4	*Human papillomavirus vaccination 2020 guideline update: American Cancer Society (ACS) guideline adaptation* Salow *et al.*, 2020^(37)^	Adaptar las recomendaciones actuales del Comité Asesor sobre Prácticas de Inmunización para la vacunación contra el Virus del Papiloma Humano.	Se recomienda la vacunación sistemática contra el Virus del Papiloma Humano entre los 9 y los 12 años para lograr vacunación oportuna. Los profesionales de la salud deben ofrecer la vacunación desde los 9 o 10 años. Se debe administrar una dosis de refuerzo a todas las personas de hasta 26 años. Los profesionales deben informar a las personas de 22 a 26 años que no se hayan vacunado. No se recomienda la vacunación de refuerzo contra el Virus del Papiloma Humano para adultos mayores de 26 años.
5	*Drivers for human papillomavirus vaccination in Valencia (Spain)* Navarro-Illana *et al.*, 2018^(38)^	Describir qué factores se asocian a la vacunación contra el Virus del Papiloma Humano en adolescentes y la opinión de sus padres/madres sobre dicha vacuna.	La cobertura de vacunación contra el Virus del Papiloma Humano es del 74,5%; los factores influyentes son el consejo del personal de salud, en especial el de enfermería, que tiene un impacto significativo sobre las otras variables. Los factores que más inciden en la vacunación son el país de origen de la madre, el estado civil de los padres y el nivel de conocimiento sobre el Virus del Papiloma Humano. Las variables a favor de la vacunación son el uso de servicios preventivos y la percepción de riesgo. Se debe fortalecer la educación y la capacitación del equipo de salud.
6	*An Integrative Review of the Influences on Decision-Making of Young People About Human Papillomavirus Vaccine* Sisson y Wilkinson, 2019^(39)^	Explorar y comprender los factores que influyen en la toma de decisiones de los jóvenes sobre la vacunación contra el Virus del Papiloma Humano.	La decisión de vacunar está influenciada por cuatro factores: el miedo, el dolor, la participación de los padres y las recomendaciones del personal de salud. Los dos últimos son determinantes para la adherencia a la vacunación, lo que refuerza el papel de las enfermeras escolares en la promoción de la vacunación contra el Virus del Papiloma Humano. Se deben generar estrategias que incluyan aspectos sociales y culturales que permitan consolidar la confianza hacia el personal de salud.
7	*Barriers to and Facilitators of Human Papillomavirus Vaccination Among People Aged 9 to 26 Years: A Systematic Review* Zheng *et al.*, 2021^(40)^	Comprender las barreras y facilitadores que enfrentan los adolescentes y jóvenes adultos de 9 a 26 años para iniciarse en la vacunación contra el Virus del Papiloma Humano.	Las barreras son la falta de conocimiento sobre el virus y la vacuna, las dudas sobre los riesgos del procedimiento, la eficacia de la vacuna, los costes, el miedo al dolor, las malas experiencias anteriores, la discriminación y la falta de tiempo. Los facilitadores son las recomendaciones del personal de salud. Fortalecer la educación y las campañas de información de la vacunación contra el Virus del Papiloma Humano puede aumentar la aceptación y mejorar las tasas de vacunación.
8	*Policy analysis of cervical cancer prevention in Iran based on the policy triangle model* Pirani *et al.*, 2023^(41)^	Analizar las políticas de prevención del cáncer cervicouterino en Irán con base en el modelo del triángulo de análisis de políticas de salud de Walt y Gilson.	Los principales problemas de la política de vacunación son la falta de supervisión, evaluación, uso de la evidencia científica, debilidades en la estructura organizativa, la coordinación entre los distintos sectores y la gestión ejecutiva. También se observan deficiencias en la aplicación de principios científicos, protocolos operativos y uso de tecnologías. Se requiere fortalecer la prevención, educación y formación profesional para la toma de decisiones en salud pública.
9	*The feasibility of universal HPV vaccination program in Shenzhen of China: a health policy analysis* Chen y Wong, 2019^(42)^	Evaluar la viabilidad de un programa universal de vacunación contra el Virus del Papiloma Humano en adolescentes mujeres para buscar estrategias para la formulación de políticas en Shenzhen, China.	Existe un interés creciente en la vacunación contra el Virus del Papiloma Humano, favorecido por condiciones de vida favorables en Shenzhen. Persisten desafíos en la coordinación entre instituciones de salud, movilidad política, planes y estrategias para la vacunación y compromiso por parte de actores relevantes como padres y Organizaciones de la Sociedad Civil.
10	*Human papillomavirus (HPV) vaccination in a privately funded program in Ghana: A qualitative case study* Marfo *et al.*, 2024^(43)^	Identificar los procesos de vacunación contra el Virus del Papiloma Humano, los desafíos de la práctica y los intereses de las políticas.	La falta de una política que guíe el programa de vacunación afecta a su eficiencia. Se reconoce como limitación el énfasis en la historia sexual y el cribado del Virus del Papiloma Humano como medio para tomar la decisión de vacunar, asociado a la falta de programas formales de educación sobre el Virus del Papiloma Humano para el equipo de salud y los receptores. Además, falta apoyo por parte de los líderes para impulsar estos programas.
11	*Implementing the free HPV vaccination for adolescent girls aged below 14 in Shenzhen, Guangdong Province of China: experience, challenges, and lessons* Wu *et al.*, 2023^(44)^	Evaluar un proyecto piloto en Shenzhen, Provincia de Guangdong, para promover la inclusión de la vacuna contra el Virus del Papiloma Humano en el programa local de inmunización y abordar las barreras existentes para su implementación.	El aumento de la vacunación fue posible gracias al compromiso del Gobierno, la financiación, la colaboración entre los diferentes sectores y la garantía de acceso a esta prestación. Aún quedan desafíos por superar, como lograr la cobertura universal, hacer frente al rechazo de la vacunación por parte de algunos grupos y ampliar el rango de edad para que más personas puedan acceder a ella. El éxito depende de la voluntad política de los Estados para fortalecer las políticas públicas de salud.
12	*Prevalence of high-risk human papillomavirus among women in two English-speaking Caribbean countries* Andall-Brereton, 2017^(45)^	Caracterizar las infecciones por Virus del Papiloma Humano de alto riesgo en una muestra de mujeres en dos pequeños países caribeños de habla inglesa: Saint Kitts y Nevis y San Vicente y las Granadinas.	Se identificaron 12 genotipos de Virus del Papiloma Humano de alto riesgo en los países del Caribe anglófono, donde la prevalencia de esta infección es alta. Las infecciones por Virus del Papiloma Humano se asociaron con una menor edad, una mayor frecuencia de citologías anormales y una mayor paridad. Más del 20% de las mujeres presentó múltiples infecciones, lo que supone un mayor riesgo citológico. Estos resultados respaldan la implementación de la vacuna nonavalente en los programas escolares.
13	*Public health impact and cost effectiveness of routine and catch-up vaccination of girls and women with a nine-valent HPV vaccine in Japan: a model-based study* Cody *et al.*, 2021^(46)^	Evaluar el impacto en la salud de la población y la relación coste-beneficio de la vacunación de rutina de niñas y mujeres de 11 a 26 años de edad con vacunas contra el Virus del Papiloma Humano tetravalentes o nonavalentes en Japón, en comparación con ninguna vacunación.	Se proyecta que la vacuna nonavalente tendrá un gran impacto en la salud pública de Japón, ya que reduciría en más del 86% los casos de cáncer cervicouterino, lo que evitaría más de 450.000 casos nuevos y más de 50.000 muertes. También tendría un impacto en las verrugas genitales y otros cánceres relacionados con el Virus del Papiloma Humano. La vacuna nonavalente, con campañas dirigidas a los adolescentes, es efectiva para prevenir el cáncer cervicouterino. Se debe ampliar los programas de vacunación y hacerlos una prioridad de salud pública.
14	*Public health impact and cost-effectiveness of a nine-valent gender-neutral HPV vaccination program in France* Majed *et al.*, 2021^(47)^	Evaluar el impacto en la salud pública y la rentabilidad de la vacunación no sexista en comparación con un programa de vacunación solo para niñas.	La vacunación neutral logró reducciones adicionales en incidencia y mortalidad de cánceres relacionados con el Virus del Papiloma Humano. También se previnieron otras enfermedades relacionadas al Virus del Papiloma Humano. Un escenario con 60% de cobertura en ambos sexos redujo significativamente la carga de enfermedad, pero aumentó el *Incremental Cost-Effectiveness Ratio* a €40.401. La inclusión de niños demostró beneficios en salud pública y respaldó la ampliación del programa de vacunación para incluir a ambos sexos con la vacuna nonavalente.
15	*Public health impact of 2-, 4-, and 9-valent HPV vaccination in females on cervical and noncervical diseases in men and women under different coverage scenarios in China: A simulation study* Diakite *et al.*, 2023^(48)^	Estimar el impacto en la salud pública en China de las estrategias de vacunación contra el Virus del Papiloma Humano (con y sin protección cruzada), con vacunas bivalentes, tetravalentes y nonavalentes.	Todas las estrategias de vacunación generan un impacto positivo en la reducción del cáncer cervicouterino y otras enfermedades asociadas al Virus del Papiloma Humano. La vacuna nonavalente muestra mayor efectividad, proyectando la disminución de hasta 5 millones de casos en 100 años. Se demostró que la vacunación primaria, con cualquier tipo de vacuna contra el Virus del Papiloma Humano, es el factor principal para disminuir la carga de esta enfermedad.
16	*Toward universal human papillomavirus vaccination for adolescent girls in Hong Kong: a policy analysis* Chen *et al.*, 2020^(49)^	Examinar la participación y las interacciones de las partes interesadas para promover un programa universal de vacunación contra el Virus del Papiloma Humano, utilizando el marco del triángulo de análisis de políticas de salud de Walt y Gilson para el análisis estructurado de políticas de salud.	El sistema político, junto con los valores propios de su cultura y la influencia de Occidente, se relaciona en cómo las personas perciben la creación de políticas de vacunación contra el Virus del Papiloma Humano. Identifico posturas éticas diversas y uso desigual de la evidencia científica. Para avanzar en la vacunación, se debe garantizar el acceso universal y gratuito a los adolescentes, reforzando también las estrategias que promueven la aceptación social de este tipo de procedimiento.
17	*Content analysis of digital media coverage of the human papillomavirus vaccine school-entry requirement policy in Puerto Rico* Colón *et al.*, 2021^(50)^	Analizar el contenido de la cobertura periodística digital relacionada con la implementación de la política de vacunación contra el Virus del Papiloma Humano en Puerto Rico.	La mayoría de los medios de comunicación no mencionaron el número de dosis, y el 79% la destacó como estrategia de prevención del cáncer cervicouterino. Mostraron un contenido neutral respecto a la vacuna y los cánceres asociados al Virus del Papiloma Humano. Los mensajes negativos incluían información sobre la autonomía política. Los aspectos positivos se relacionaron con la educación sexual y la prevención del cáncer. La comunicación limitada y ambivalente puede afectar a la aceptación y cobertura de la vacuna contra el Virus del Papiloma Humano.
18	*Awareness of human papillomavirus and acceptability of the vaccine among women in Palestine: is it time for policy adjustment?* Elshami *et al.*, 2022^(51)^	Evaluar el nivel de conocimiento de las mujeres palestinas sobre el Virus del Papiloma Humano, así como el nivel de aceptación y conocimiento de la vacuna y los factores asociados a estos.	El conocimiento sobre el Virus del Papiloma Humano y su vacuna fue extremadamente bajo. Solo un 8,1% de las mujeres habían escuchado acerca del Virus del Papiloma Humano, y solo 46 mujeres de este grupo conocían acerca de la vacuna. La mayoría expresó su disposición para vacunarse o vacunar a sus hijas, siempre y cuando la vacuna fuera gratuita o tuviera un copago asequible. La incorporación de la vacuna contra el Virus del Papiloma Humano en un programa nacional puede mejorar la aceptación y el conocimiento.
19	*Health policy analysis of the non-implementation of HPV vaccination coverage in the pay for performance scheme in France* Ohannessian *et al.*, 2019^(52)^	Analizar las razones de la no implementación de indicador para la vacuna contra el Virus del Papiloma Humano en el Plan Nacional de Control del Cáncer en Francia.	Los actores de la política pública fueron el Ministerio de Salud y el Instituto del Cáncer Francés, que la formularon; el seguro público, que fue su objetivo indirecto, y los médicos, que la ejecutaron directamente. La política pública careció de fundamentos basados en la evidencia y no se tuvo en cuenta en el informe del Plan Nacional de Control del Cáncer. El contexto se vio marcado por la resistencia a las vacunas y las preocupaciones éticas de la atención primaria, en oposición al Ministerio de Salud. El proceso incluyó a múltiples partes interesadas con una gobernanza compleja. Estas condiciones pudieron contribuir al fracaso de la política pública de vacunación contra el Virus del Papiloma Humano en Francia.
20	*Implementation of the human papillomavirus school-entry requirement in Puerto Rico: barriers and facilitators using the consolidated framework for implementation research* Colón-López *et al.*, 2021^(53)^	Identificar las barreras y facilitadores para la implementación de la vacuna contra el Virus del Papiloma Humano en estudiantes de 11 a 12 años en Puerto Rico, utilizando el marco consolidado para la investigación de implementación.	El facilitador debe conocer las necesidades de los padres y estudiantes para cumplir con la vacunación antes del ingreso escolar. Los padres apoyaron la implementación de iniciativas de política pública que respaldaran otras estrategias de vacunación. Las barreras fueron el coste de la administración de la vacuna en proveedores privados, la influencia de las redes sociales y la escasez de enfermeras escolares como parte del personal clave.
21	*Evolution of Public Health Human Papillomavirus Immunization Programs in Canada* Goyette *et al.*, 2021^(54)^	Describir la evolución de estos programas de vacunación en Canadá.	La vacunación en niñas en edad escolar comenzó en los años escolares 2007/08 a 2010/11 con tres dosis de la vacuna tetravalente contra el Virus del Papiloma Humano en todas las provincias, excepto en Quebec. La mayoría de las provincias incluyen vacunación de rescate y vacunación para grupos de alto riesgo. Las tasas de vacunación varían notoriamente entre provincias; solo en Isla Príncipe Eduardo superan el 80%.

Fuente: Elaboración propia.

### Componentes Centrales del Triángulo de Walt y Gilson

Como actores sanitarios de primera línea, los profesionales de la salud en general y de enfermería en particular están en una posición privilegiada para aumentar la aceptación ciudadana de los procesos de vacunación, cobertura y acceso. Respecto a contenido, contexto y proceso, se necesitan nuevas estrategias para ampliar la aceptación del proceso dada la presencia de entornos dinámicos y formular políticas de vacunación eficaces, basadas en la evidencia (Cuadro 2).

**Cuadro 2:** Componentes del triángulo de Walt y Gilson, autores y elementos teóricos distintivos que conforman los artículos obtenidos en la revisión estructurada e informes relacionados con la implementación de políticas – Santiago, Chile, 2025.

Componente	Autores	Elementos distintivos
Actores	Lin *et al.*, 2022^(34)^	Los profesionales de la salud son protagonistas de los procesos de vacunación contra el Virus del Papiloma Humano. Sin embargo, los profesionales de enfermería están en una posición privilegiada para fomentar la aceptación de la vacuna contra el Virus del Papiloma Humano por parte de la ciudadanía, representando un actor clave para aumentar la cobertura y acceso. En ello, organismos nacionales e internacionales ejercen influencias en los procesos de implementación de esta política pública.
	Chiu, 2021^(35)^	
	Ebu *et al.*, 2021^(36)^	
	Salow *et al.*, 2020^(37)^	
	Navarro *et al.*, 2018^(38)^	
	Sisson *et al.*, 2019^(39)^	
	Zheng *et al.*, 2021^(40)^	
	Pirani *et al.*, 2023^(41)^	
Contenido	Chen *et al.*, 2019^(42)^	Para una política eficaz de vacunación contra el Virus del Papiloma Humano y la promoción de la vacunación universal y gratuita contra el Virus del Papiloma Humano, se necesitan nuevas estrategias para ampliar la aceptación del proceso, formular políticas basadas en hechos y valores, conectar la investigación con la formulación de políticas y mejorar la formación de coaliciones público-privadas.
	Marfo *et al.*, 2024^(43)^	
	Wu *et al.*, 2023^(44)^	
	Pirani *et al.*, 2023^(41)^	
	Andall-Brereton *et al.*, 2017^(45)^	
	Cody *et al.*, 2021^(46)^	
	Majed *et al.*, 2021^(47)^	
	Diakite *et al.*, 2023^(48)^	
Contexto	Chen *et al.*, 2019^(42)^	A pesar de los avances, el cáncer cervicouterino sigue afectando a las personas que se desenvuelven en contextos diferentes y entornos dinámicos. Se recomienda financiar programas de prevención, sensibilizar a la población, educar en autocuidado y ajustar la formación universitaria, ya que son estrategias clave para fortalecer la detección y apoyar la adopción de decisiones en políticas públicas de salud basadas en la evidencia.
	Marfo *et al.*, 2024^(43)^	
	Wu *et al.*, 2023^(44)^	
	Chen *et al.*, 2020^(49)^	
	Colón *et al.*, 2021^(50)^	
	Elshami *et al.*, 2022^(51)^	
	Ohannessian *et al.*, 2019^(52)^	
	Navarro *et al.*, 2018^(38)^	
	Colón *et al.*, 2021^(53)^	
	Zheng *et al.*, 2021^(40)^	
	Pirani *et al.*, 2023^(41)^	
	Andall-Brereton *et al.*, 2017^(45)^	
	Sisson *et al.*, 2019^(39)^	
Proceso	Chen *et al.*, 2019^(42)^	Desde su introducción, los programas de vacunación contra el Virus del Papiloma Humano en Chile y el mundo han evolucionado esquemas y dosis. En ello, es necesario afrontar los retos para lograr la cobertura universal, que incluyen abordar la reticencia a la vacunación, ampliar el programa para abarcar un rango de edad más amplio y garantizar la calidad constante de los servicios de vacunación en Atención Primaria de Salud. Superar estos retos requerirá estrategias innovadoras, colaboraciones público-privadas, financiación, estrategias sostenibles y desarrollar directrices estandarizadas para respaldar la práctica de la vacunación contra el Virus del Papiloma Humano basada en la evidencia.
	Marfo *et al.*, 2024^(43)^	
	Wu *et al.*, 2023^(44)^	
	Pirani *et al.*, 2023^(41)^	
	Goyette *et al.*, 2021^(54)^	
	Elshami *et al.*, 2022^(51)^	
	Ohannessian *et al.*, 2019^(52)^	
	Colón *et al.*, 2021^(53)^	
	Lineamientos técnicos-operativos de la vacunación escolar 2025^(55)^	
	Ord. N°. 988: Informa “*catch-up*” de vacunación contra el Virus del Papiloma Humano, Chile, 2025^(56)^	
	Orden N.º 1032 (B27N°): Informes de actualización de la vacuna contra el VPH para estudiantes del 5° básico, Chile, 2025^(57)^	

Fuente: elaboración propia.

## DISCUSIÓN

El desarrollo de nuestro proceso de investigación nos permitió examinar y analizar la política de vacunación contra el VPH implementada en Chile desde la perspectiva TWG, y definir aspectos a mejorar, prestando siempre atención al contenido, los contextos, los procesos y los participantes de esta política que se ubican en el centro del modelo. Este enfoque de análisis ascendente o *bottom-up* permitió obtener una visión más amplia de la PP, ya que permite analizar los detalles y componentes específicos de esta.

### Los Actores

En el modelo TWG, los actores son identificados como individuos y organizaciones que actúan e influyen en una PP. En la política de vacunación contra el VPH, tanto las personas que reciben la vacuna como quienes disponen de este procedimiento clínico tienen un papel fundamental, ya que esta medida preventiva es más que una simple decisión técnica adoptada por un comité científico y los departamentos de salud, y aplicada por profesionales y recepcionada por la población, ya que representa una estrategia de salud pública clave para intervenir sobre la incidencia de una de las principales causas de muerte entre las mujeres: el CaCu asociado al VPH^(58)^.

Lin et al.^(34)^ sostienen que los profesionales de enfermería son protagonistas de la vacunación y están en una posición privilegiada para fomentar la aceptación de la vacuna contra el VPH, por lo que son actores clave para aumentar la cobertura de vacunación. Estos profesionales de primera línea implementan y reestructuran las PPs, lo que tiene un impacto fundamental en los resultados en salud^(59)^. Sisson y Wilkinson^(39)^ afirman que los profesionales de enfermería deben considerar que la decisión de los jóvenes respecto a la vacunación contra el VPH está influenciada por múltiples factores, incluidos el miedo, la percepción del riesgo, el dolor, la implicación de los padres y la propia influencia de los profesionales de la salud. Sin embargo, precisamente para fortalecer la cobertura sanitaria y la justicia social, el estudio de Chiu de 2021^(35)^ afirma que es importante que la enfermería desarrolle conocimientos y liderazgo político para fortalecer los sistemas de salud y las prioridades globales. En lo que respecta a la mejora de los procesos de vacunación contra el VPH, Lin et al.^(34)^, Ebu et al.^(36)^, Salow et al.^(37)^ y Navarro-Illana et al.^(38)^coinciden en que, por un lado, se necesita apoyo institucional para desarrollar programas de educación sanitaria dirigidos a los profesionales de enfermería como agentes promotores de la vacuna contra el VPH, y, por otro, se deben fortalecer los conocimientos y actitudes sobre el VPH y eliminar el estigma que genera hablar sobre temas relacionados con el VPH con las personas.

En complemento, entre las personas que reciben este procedimiento clínico, las recomendaciones de los proveedores de atención sanitaria representan importantes facilitadores, siendo un estímulo clave para la decisión de recibir la vacuna tanto para adolescentes como para jóvenes adultos^(34)^. En este sentido, Navarro-Illana et al.^(38)^, Sisson y Wilkinson^(39)^, Zheng et al.^(40)^ y Pirani et al.^(41)^ enfatizan en que, frente a las barreras percibidas por la ciudadanía para la vacunación contra el VPH, que incluyen principalmente la falta de conocimiento, preocupaciones sobre la seguridad y la eficacia de la vacuna, problemas relacionados con el coste, miedo al dolor, experiencias negativas previas con vacunas, discriminación y falta de tiempo, la educación y las campañas para aumentar el conocimiento sobre la susceptibilidad y la transmisión del VPH, además de aclarar la seguridad y la efectividad de la vacuna, son las estrategias que pueden promover la aceptación y el aumento de las tasas de vacunación.

### El Contenido

En el TWG, lo operativo que involucra el contenido incluye objetivos de políticas, directrices, reglamentos, acciones concretas y lineamientos que favorecen el proceso de implementación. En ello, este “qué hacer” de las instituciones moviliza recursos y determinadas estrategias que pueden fortalecer los resultados en salud que se persiguen. De esta manera, la eficacia de la política de vacunación contra el VPH, la promoción de la salud y la prevención de enfermedades potencialmente mortales requieren nuevas estrategias para ampliar la aceptación del proceso, formular políticas basadas en hechos y valores colectivos, conectar la investigación con la formulación de políticas y mejorar la formación de coaliciones público-privadas. En este sentido, el estudio de Chen y Wong^(42)^ de 2019 concluye que, para lograr mayores estándares preventivos de CaCu, se deben implementar procesos de vacunación sistemática contra el VPH entre los 9 y los 12 años de edad, así como refuerzos contra el VPH para todas las personas de hasta 26 años que no hayan sido adecuadamente vacunadas. Para mayores de 26 años, no recomiendan la vacunación de refuerzo contra el VPH, dado que representa una estrategia sanitaria menos eficaz para reducir el riesgo de CaCu.

Para Marfo et al.^(43)^, si bien muchos vacunadores abogan por un programa universal de vacunación contra el VPH, algunos responsables de políticas y programas se muestran reacios a priorizar su promoción. Esto genera limitaciones en estrategias preventivas, pues un programa de vacunación sin una política puede verse limitado en calidad y eficiencia, ya que no habrá medidas de rendición de cuentas ni sostenibilidad. Es ahí donde se precisan directrices estandarizadas y PPs para respaldar la práctica de la vacunación contra el VPH basada en la evidencia. Pirani et al.^(41)^ también enfatizan la necesidad de fortalecer PPs que apoyen estos procesos sanitarios, complementando con la necesidad de financiar programas de prevención, sensibilizar a la población, educar en autocuidado y ajustar la formación universitaria, pues representan estrategias clave para fortalecer la detección y apoyar decisiones en PPs de salud.

Respecto al fortalecimiento de los procesos sanitarios para vigorizar la estrategia de vacunación contra el VPH, Wu et al.^(44)^en 2023 no solo abordan la reticencia a la vacunación a través de acciones de concienciación pública, educación y comunicación sanitaria sobre la vacuna contra el VPH desde la atención primaria, sino que también enfatizan que, para afrontar los retos de la cobertura universal de la vacunación, se requiere un firme compromiso gubernamental y voluntad política, financiación suficiente, colaboración multisectorial público-privada y garantía de accesibilidad y asequibilidad de las vacunas. Por otro lado, Andall-Brereton et al.^(45)^, Cody et al.^(46)^, Majed et al.^(47)^ y Diakite et al.^(48)^ complementan respecto a evidencia clave sobre la prevalencia del VPH y sus factores de riesgo, afirmando que es necesario cambiar de la vacunación bivalente e incorporar de modo urgente la vacuna nonavalente en los programas de inmunización contra el VPH, aprovechando los sistemas de salud escolar, pues evitará un número significativo de casos de enfermedades relacionadas con el VPH tanto en hombres como en mujeres.

### El Contexto

A pesar de los avances en cobertura de vacunación contra el VPH, las condiciones sociales, económicas, políticas, culturales y ambientales afectan a la PP, provocando que el CaCu relacionado siga afectando a personas en todas las regiones del mundo. En ello, la literatura es enfática en señalar que sensibilizar a la población, educar en autocuidado y ajustar la formación universitaria, para que los nuevos profesionales de la salud impacten sobre las personas en estas materias, resultan ser estrategias clave para fortalecer la promoción de la salud, prevención de enfermedades asociadas, fortalecer la detección y apoyar la implementación de PPs de salud en los territorios.

Para Chen y Wong^(42)^ de 2019 y Marfo et al.^(43)^, la calidad y la eficiencia de los programas de vacunación contra el VPH requieren una comprensión profunda de las características de los grupos destinatarios de esta medida sanitaria, ya que esto permite respaldar las políticas de vacunación contra el VPH basándose en la evidencia. En complemento, para Wu et al.^(44)^, Chen et al.^(49)^ en 2020 afirman que la sostenibilidad de la vacunación contra el VPH dependerá de la voluntad política para incorporar intervenciones preventivas basadas en hechos y valores en la política, teniendo en cuenta los factores contextuales que puedan influir en su implementación en diferentes entornos.

Las estrategias de comunicación y la información disponible para las comunidades también son motivo de preocupación. Según Colón-López et al.^(50)^, los temas neutrales y negativos sobre el VPH podrían influir en la preocupación pública con respecto a las tasas de vacunación contra el VPH. Por otro lado, Elshami et al.^(51)^ planteaban cómo el bajo nivel de conocimiento general sobre el VPH afecta a la vacunación contra este virus. Ambas situaciones pueden influir en la gobernanza de las políticas de vacunación y contribuir a conformar contextos desfavorables para la política, según Ohannessian et al.^(52)^. No obstante, para Navarro-Illana et al.^(38)^, Sisson y Wilkinson^(39)^, Colón et al.^(50)^ en 2021, Zheng et al.^(40)^ y Pirani et al.^(41)^, la mayoría de las barreras para esta política pueden superarse mediante la implementación de programas educativos y de capacitación en las escuelas, la sensibilización de la población y la educación en autocuidado, que pueden apoyar esta política de salud, como enfatizan Andall-Brereton et al.^(45)^, y facilitar su aceptación y mejorar las tasas de inmunización contra el VPH.

### El Proceso

Desde su introducción, los programas de vacunación contra el VPH han evolucionado en esquemas y dosis para abordar desafíos de cobertura, en diversidades territoriales y sociales, mediante diferentes estrategias de desarrollo e implementación de esta PP. La literatura analizada enfatiza que superar estos retos requerirá estrategias de vacunación contra el VPH basadas en la evidencia que identifiquen grupos de edades sobre quienes la vacunación reporte mayores indicadores de eficacia y eficiencia sanitaria en materia de prevención de enfermedades asociadas, tal como lo sugiere Chen et al. en 2019^(42)^, Marfo et al.^(43)^, Wu et al.^(44)^ y Goyette et al.^(54)^.

En materia de evaluación, se deben realizar adaptaciones, modificaciones y ajustes al proceso de implementación de esta PP. Colón-López et al.^(50)^, Pirani et al.^(41)^, Elshami et al.^(51)^ y Ohannessian et al.^(52)^ afirman que el fortalecimiento de programas educativos para la ciudadanía en términos de sensibilizar a la población en estas materias podría garantizar estrategias sostenibles de cobertura, acceso y oportunidad de esta estrategia sanitaria.

En este contexto, uno de los grandes desafíos de los programas de vacunación contra el VPH es que cuenten con estrategias que permitan la sostenibilidad de la PP en el tiempo, logrando coberturas sanitarias en las poblaciones objetivo. Un ejemplo de esto constituye las políticas de vacunación en Chile, las cuales están garantizadas por ley mediante la vacuna nonavalente VPH^(17)^. Estas garantías requieren estrategias como el acercamiento de los profesionales de salud a la comunidad, mediante campañas en centros escolares^(55)^, o rescate de grupos rezagados^(56,57)^, con el fin de alcanzar la cobertura objetivo. El éxito de una iniciativa tan trascendental como la prevención y eventual erradicación del CaCu requiere el compromiso del equipo de salud y un respaldo sostenido del Estado para garantizar equidad y oportunidad en beneficio de la población.

Las limitaciones mantenidas en este estudio se relacionan con el difícil acceso a informes oficiales de evaluación de los procesos de implementación de esta política en territorio nacional. Del mismo modo, la escasez de estudios en latinoamérica plantea desafíos para equipos de investigadores y gobiernos frente a la necesidad de transitar hacia un desarrollo humano integral y sostenible.

## CONCLUSIÓN

El presente estudio comprobó que el análisis de PPs mediado por el TWG permite elaborar una caracterización cabal de la PP sanitaria, pues identifica las interacciones entre contexto, contenido y proceso, y cómo los actores resultan ser protagonistas del modelo. Desde un punto de vista *bottom-up*, cada componente del TWG evidencia que la PP es dinámica y requiere la adaptación continua a las realidades sociales y a las necesidades de las personas. Si bien la vacunación contra el VPH representa una de las intervenciones más seguras y eficaces contra los cánceres relacionados con el virus tanto en Chile como en otras comunidades, el papel de los profesionales de la salud en general, y de enfermería en particular, exige una participación activa en todas las fases del ciclo de la PP, desde el diseño y la toma de decisiones hasta la implementación y evaluación de estas. Para ello, su capacidad de liderazgo, su cercanía con la comunidad a través del vínculo estratégico entre las necesidades de la población y las garantías que ofrece el Estado, y su visión integral del cuidado de las personas posicionan a esta disciplina como un agente clave en la promoción de estrategias como la vacunación contra el VPH, lo que asegura que el derecho a la salud se materialice en los grupos más vulnerables. Esta es una prioridad global que aspira a un desarrollo humano integral y sostenible.

## Data Availability

Todo el conjunto de datos que apoyan los resultados de este estudio fue publicado en el propio artículo.
